# Biosynthesis of Biphenomycin-like Macrocyclic Peptides by Formation and Cross-Linking of *Ortho*-Tyrosines

**DOI:** 10.1021/jacs.5c06044

**Published:** 2025-06-26

**Authors:** Chandrashekhar Padhi, Lingyang Zhu, Jeff Y. Chen, Chuan Huang, Ryan Moreira, Gregory L. Challis, Max J. Cryle, Wilfred A. van der Donk

**Affiliations:** † Department of Chemistry and Howard Hughes Medical Institute, 14589 University of Illinois at Urbana−Champaign, 600 South Mathews Avenue, Urbana, Illinois 61801, United States; ‡ School of Chemical Sciences NMR Laboratory, University of Illinois at Urbana−Champaign, Urbana, Illinois 61801, United States; § Carl R. Woese Institute for Genomic Biology, University of Illinois at Urbana−Champaign, 1206 West Gregory Drive, Urbana, Illinois 61801, United States; ∥ Department of Biochemistry and Molecular Biology, Biomedicine Discovery Institute, 2707 Monash University, Clayton, Victoria 3800, Australia; ⊥ ARC Centre of Excellence for Innovations in Peptide and Protein Science, Biomedicine Discovery Institute, Monash University, Clayton, Victoria 3800, Australia; # Department of Chemistry, University of Warwick, Coventry CV4 7AL, UK

## Abstract

Ribosomally synthesized and posttranslationally modified peptides (RiPPs) are a growing class of natural products. Multinuclear nonheme iron-dependent oxidative enzymes (MNIOs, previously DUF692) are involved in a range of unprecedented biochemical reactions. Over 13,500 putative MNIO-encoding biosynthetic gene clusters (BGCs) have been identified by sequence similarity networks. In this study, we investigated a set of precursor peptides containing a conserved FHAFRF motif in MNIO-encoding BGCs. These BGCs contain genes encoding an MNIO, a RiPP recognition element-containing protein, an arginase, a hydroxylase, and a vitamin B12-dependent radical SAM enzyme (B12-rSAM). Using heterologous reconstitution of a representative BGC from (*pbs* cluster) in , we demonstrated that the MNIO in conjunction with the partner protein catalyzes *ortho*-hydroxylation of each of the phenylalanine residues in the conserved FRF motif, the arginase forms an ornithine from the arginine, the ornithine residue is hydroxylated, and the B12-rSAM cross-links the *ortho*-Tyr side chains by a C–C linkage forming a macrocycle. A protease matures the RiPP to its final form. The elucidated structure shares close similarity to biphenomycins, a class of peptide antibiotics for which the biosynthetic pathway has not been characterized. Substrate scope studies suggest some tolerance of the MNIO and the B12-rSAM enzymes. This study expands the diverse array of posttranslational modifications catalyzed by MNIOs and B12-rSAM enzymes, deorphanizes biphenomycin biosynthesis, and provides a platform for the production of analogs from orthologous BGCs.

## Graphical abstract

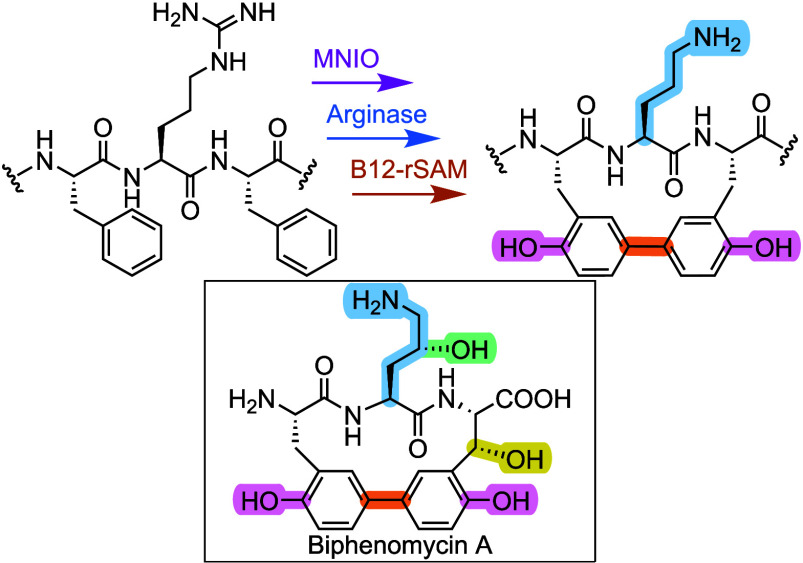

## Introduction

The use of biocatalysis in industry has witnessed a remarkable rise with most pharmaceutical companies having programs to use enzymes in especially the production phase of drug development.^1–4^ Enzymes decrease step-count of synthetic routes, provide high stereoselectivities, and result in more cost-effective and greener manufacturing.^5–9^ However, a key limitation is the repertoire of available enzymes. Whereas a process chemist benefits from a century of synthetic chemistry methods to devise various potential routes to a drug candidate, retrosynthetic biosynthesis^10^ is at present much more limited. Directed evolution has proven remarkably successful in improving low enzyme activities to commercially useful levels,^11^ but if a given transformation has no precedent in enzyme catalyzed reactions, the process of finding a starting point for such evolution is challenging. One source of new enzymes is the microbial genomes that encode a myriad of proteins with unknown function, especially those involved in natural product biosynthesis,^12^ but assignment of their catalytic power is difficult because their substrates are unknown and often not readily accessible.

Natural products (NPs) and NP-derived molecules exhibit tremendous chemical and structural diversity and serve as a major source of modern therapeutics^13–15^ and novel enzymes.^12^ One class of NPs is the family of ribosomally synthesized and posttranslationally modified peptides (RiPPs)^16^ that contain intriguing chemical functionalities that confer various biomedically relevant activities such as antibiotic, antiviral, and protease inhibitory activities.^17–19^ Enzymes that introduce these functionalities are typically encoded by genes near the gene encoding the RiPP precursor peptide(s) in biosynthetic gene clusters (BGCs). This proximity of the substrate gene provides a key advantage for assigning function to proteins of unknown function.^20^ Within each RiPP family, the types of modifications are numerous and include methylation, epimerization, peptide skeleton rearrangements, and macrocyclization.^16^ Classical approaches in NP discovery typically employ chemical extraction and rely on producer cultivation and NP production. However, recent advancements in (meta)­genomic sequencing techniques have resulted in the detection of novel RiPP BGCs in genomes of uncultivated bacteria, thus expanding the chemical space of NPs.^21,22^ Since RiPPs are ribosomally produced and modified by enzymes, transplanting these genomic elements from an uncultivated organism into a heterologous laboratory strain has greatly accelerated the discovery of novel RiPP families,^16^ and thereby their biosynthetic enzymes.^20^

A recently characterized family of RiPP modification enzymes are the multinuclear nonheme iron-dependent oxidative enzymes (MNIOs), formerly known as domain of unknown function 692 (DUF692). MNIOs catalyze diverse posttranslational chemistries^23^ (Figure 1) such as oxazolone and thioamide formation in methanobactins,^24^ carbon excision in thiaglutamate,^25^ generation of thiooxazoles in bufferins,^26^ formation of two rings in chryseobasins,^27^ C-terminal amidation in methanobactin analogs,^28^ and the transformation of an Asp residue into a C-terminal α-keto acid in pyruvatides.^29^ Given the highly diverse reactions, at present, prediction of the outcome of MNIO-catalyzed transformations on precursor peptides is not possible for MNIOs that are not highly homologous to proteins that have been previously investigated.

**Figure 1:**
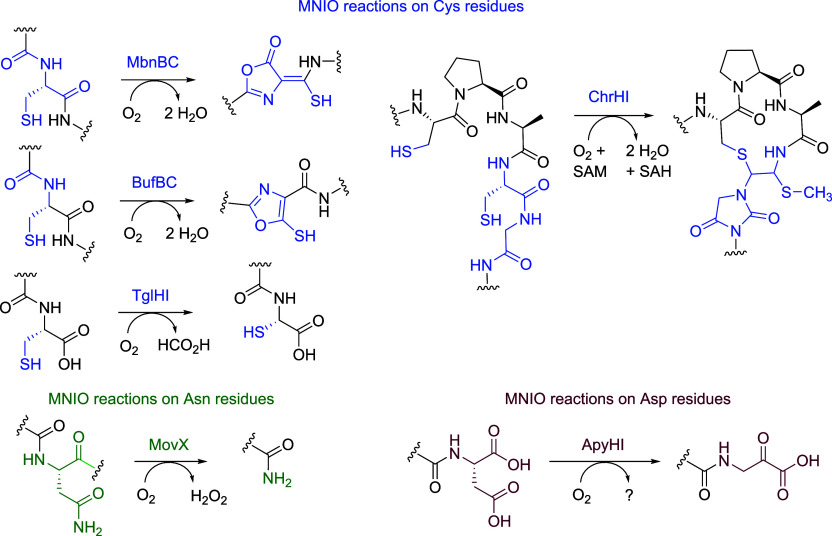
MNIO catalyzed reactions. The majority of known MNIO reactions utilize Cys residues as the substrate (in blue). Only two noncysteine-based reactions acting on Asn (green) and Asp (maroon) residues have been reported to date.

In this study, we generated a sequence similarity network (SSN) for over 13,500 MNIOs using the Enzyme Function Initiative (EFI) tools,^30,31^ and extracted the harboring BGCs using the RODEO program.^32^ We then analyzed the precursor sequences to identify putative RiPP precursor peptides sharing a conserved motif. Based on this approach, putative precursors with conserved FHAFRF-, FHTFMF-, and YHx_1_Yx_2_Y-motifs (x_1_ = S/T/A; x_2_ = T/V/A) were identified. The corresponding BGCs contain genes encoding an MNIO, a hypothetical partner protein for the MNIO, and a B12-dependent rSAM. In certain cases, multiple B12-rSAM-encoding genes were detected, while an arginase- and a cupin-encoding gene was only observed in BGCs containing the FHAFRF-motif precursor. Using pathway reconstitution in as a heterologous host, the modified products of the FHAFRF motif-containing precursor from BE23 (*pbs* cluster) were structurally characterized using a combination of analytical chemistry techniques. The MNIO (PbsC) partners with a RiPP recognition element-containing^33^ protein (PbsD) to introduce *ortho-*hydroxylations on the two aromatic residues of the FRF-part of the conserved motif, followed by the deguanidination of the Arg to Orn catalyzed by the arginase (PbsE), a transformation that took place exclusively on the bis-hydroxylated precursor peptide. Next, the cupin-like hydroxylase (PbsQ) hydroxylated the newly formed Orn residue. Subsequently, the B12-rSAM (PbsB) cross-linked the hydroxylated aromatic residues via a C–C linkage. The macrocyclic product is ultimately processed by the TldD type HExxxH motif containing protease (PbsP), thus releasing the final RiPP harboring an N-terminal macrocyclic ring. The modified product shares structural similarity with biphenomycins, a class of biaryl-linked peptide antibiotics for which the biosynthetic pathway had remained unknown.^34–39^ We assessed the substrate tolerance of the MNIO PbsC and the B12-rSAM PbsB towards a series of FHAFRF-variants including motifs containing tyrosines at the modification sites that were identified in orthologous BGCs.

In addition to the studies in , the activity of the MNIO and the arginase enzymes of the *pbs* cluster were reconstituted in vitro, and utilizing AlphaFold models, we predict the interactions of the pathway enzymes with the precursor peptides. To our knowledge, an MNIO catalyzing hydroxylation of aromatic residues, an arginase selectively acting on a bis-hydroxylated aromatic substrate, a cupin-like protein hydroxylating an Orn residue, and a B12-rSAM introducing C–C cross-links on hydroxylated aromatic side chains, have not been described before. Overall, this study characterizes the activity of five different metalloenzymes in addition to deorphanizing the biosynthesis of the biphenomycin class of peptide antibiotics and providing a platform for production of analogs.

## Results

### Genome Mining of MNIO-Containing BGCs Using a Sequence Similarity Network

MNIOs belong to the protein family PF05114 and constitute approximately 13,500 entries in the Uniprot database as of September 2024. Utilizing the Enzyme Function Initiative Enzyme Similarity Tool (EFI-EST) version 2024_04/101,^31^ a sequence similarity network was created with default parameters and processed with an alignment score of 55 (Figure 2A). This analysis resulted in 73 clusters with at least 3 interconnected nodes. Subsequently, the Uniprot IDs of the nodes from individual clusters were extracted and subjected to RODEO analysis^32^ to obtain the genome neighborhood of the selected MNIOs. Open reading frames (ORFs) ranging from a length of 30 to 150 amino acids were extracted from the RODEO output files (a combination of multiple putative clusters and standalone ORFs) and subjected to multiple sequence alignment. The alignment was refined with two iterations. Subsequently, aligned precursors containing a conserved motif were extracted manually and curated based on their genome neighborhood.

**Figure 2:**
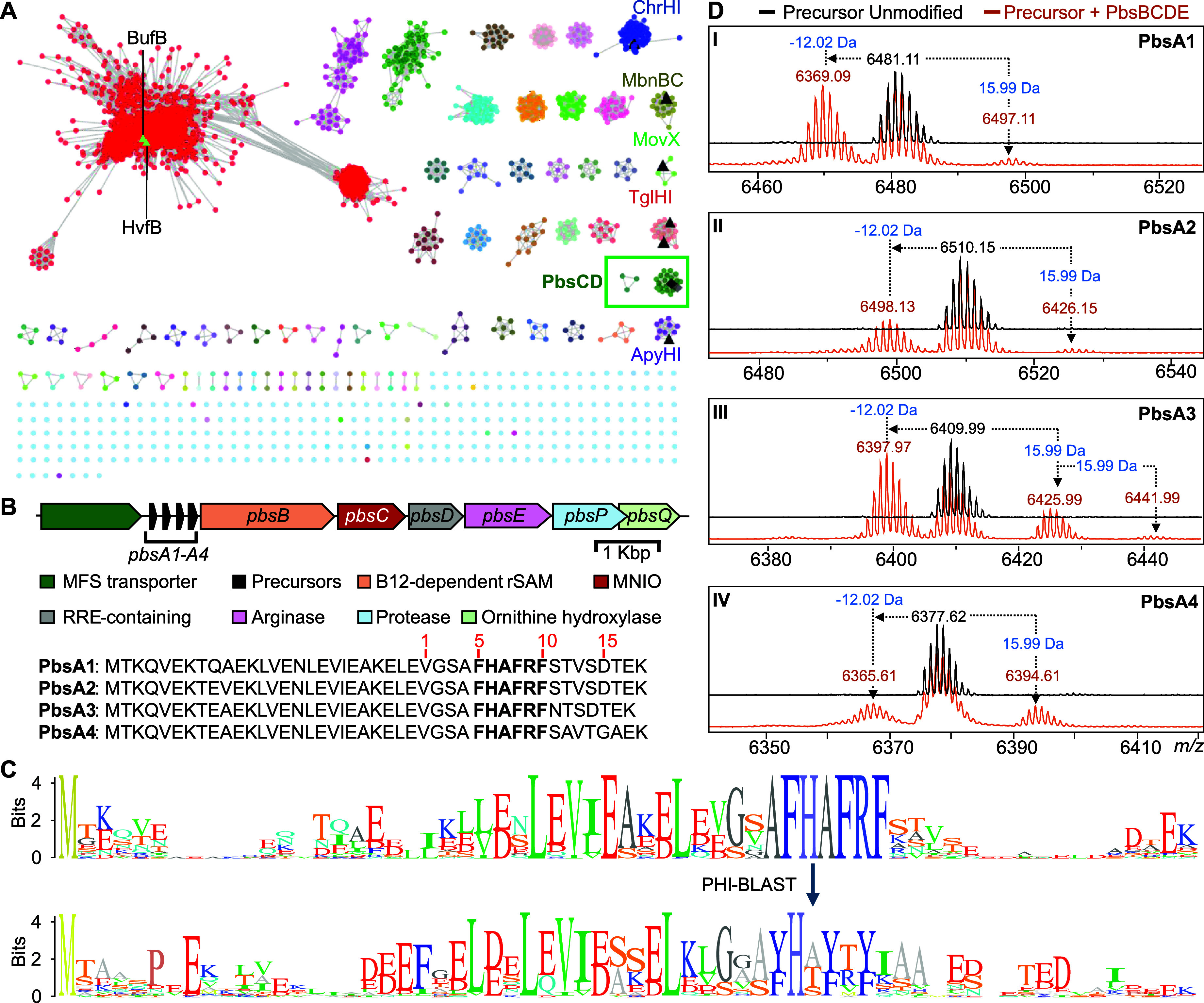
Genome mining of MNIO-containing BGCs encoding precursors with conserved motifs. (A) An SSN of ca. 13,500 MNIO homologues was generated with the EFI tools using the UNIREF90 database. RepNode 50 is shown as visualized in cytoscape v3.10. Previously characterized MNIOs are depicted in black triangles. The lime-colored triangles represent the nodes for HvfB and BufB that remain underneath the other nodes in the red cluster. The cluster boxed in green contains the MNIO PbsC that is the focus of this study. (B) The *pbs* gene cluster with the amino acid sequences of the precursors PbsA1-A4. The numbering in red is based on the GluC-digested peptide fragment isolated for further structural characterization (vide infra). (C) Sequence logo of precursor peptides containing the conserved FHAFRF motif, extracted from orthologous BGCs in the SSN (box in panel A). (D) MALDI-TOF mass spectra of N-terminally 6xHis-tagged PbsA1-A4 peptides expressed alone (black trace) or coexpressed with PbsB, PbsC, PbsD, and PbsE in (orange trace); subpanels assigned to Roman numerals I–IV for PbsA1–A4, respectively.

### Identification of BGCs Based on Conserved Motifs in the RiPP Precursor Sequence

A set of precursors containing a highly conserved FHAFRF motif was identified in over 30 potential BGCs (Figure 2C and S1). The majority of these BGCs were found in genomes of members of the phyla Actinomycetota and Bacillota. These BGCs contained genes encoding a putative MNIO, a potential partner protein for the MNIO, a putative B12-rSAM, and a tetratricopeptide repeat (TPR)-containing putative arginase (Figure 2B and S2A). We then used the conserved FHAFRF motif as a query parameter for Pattern Hit Initiated BLAST (PHI-BLAST)^40^ analysis for 12 iterations at an inclusion threshold of 0.01 to further identify 99 hits containing similar conserved motifs, i.e., FHTFMF and YHx_1_Yx_2_Y (x_1_ = S/T/A; x_2_ = T/V/A) in orthologous clusters (Figure S1). Deeper analysis of the BGCs containing the FHAFRF-, YHx_1_Yx_2_Y- and FHTFMF-motif harboring peptides indicated that the associated MNIOs of all 99 hits are part of the same cluster of enzymes in the MNIO SSN (Figure 2A; green box), suggesting they may represent isofunctional MNIOs. All orthologous clusters encoded at least one B12-dependent radical SAM (B12-rSAM) enzyme with slight variations in the remaining BGC elements (Figure S2A). For BGCs containing precursor peptides with the FHTFMF and YHx_1_Yx_2_Y motifs, the arginase was absent suggesting it may act on the Arg residue in the FHAFRF motif (Figure S2A). Conversely, a second B12-dependent rSAM and a DUF5825-containing protein was encoded adjacent to the core enzyme genes in BGCs containing the YHxYxY precursors. A recent study has characterized the function of a homologous B12-rSAM and DUF5825 pair in the biosynthesis of clavusporins,^41^ showing *C-*methylations of amino acids.

### Selection of a BGC for Enzyme Function Elucidation

We selected a BGC (*pbs* cluster) from the genome of a recently reported organism, BE23 isolated from the rhizospere of maize plants in France (GenBank: RRZF00000000.1).^42^ Previous studies have reported that strains produce siderophores that help iron uptake in potatoes, and produce bioactive molecules for inhibition of plant pathogens such as various fungi, bacteria and nematodes.^43–45^ While some antimicrobial activity has been linked to certain lipopeptides^44^ and volatile organic compounds,^46^ structural characterization of the bioactive molecules remains largely understudied. The *pbs* cluster (Figure 2B) encodes four putative precursor peptides containing an FHAFRF motif (PbsA1-A4), followed by ORFs encoding a putative B12-rSAM (PbsB), a xylose isomerase-like TIM barrel family protein with a typical signature of an MNIO (PbsC; ENA ID: RRN73831.1; RefSeq: WP_125160327), a hypothetical ORF suspected to encode a RiPP recognition element (RRE)-containing partner protein^47^ for the MNIO (PbsD) and a TPR-rich family protein belonging to the (α/β)-hydrolase superfamily believed to be an arginase (PbsE). The *pbs* cluster also contained ORFs for a transporter of the Major Facilitator Superfamily (MFS) and a putative metalloprotease PbsP (Figure S2A). When we initiated this study, these genes appeared to complete the BGC, but as discussed later, another ORF that partially overlaps with *pbsP* that was not annotated encodes for a cupin-fold protein (*pbsQ*). Since the strain of BE23 was not available in any culture collection, attempts were made to identify the product of an orthologous BGC in ATCC 21929. *B*. *clarus* was grown under a variety of growth conditions, but we did not observe production of molecules that could be linked to the BGC.

### Characterization of the Posttranslational Modifications of PbsA Peptides

For pathway reconstitution, we coexpressed different genetic elements heterologously in . Codon-optimized genes encoding the precursor peptides PbsA1-A4 were cloned into a pETDuet-1 plasmid backbone encoding a N-terminal 6xHis tag (for sequences, see Table S1). Codon-optimized ORFs for PbsB, PbsC, PbsD and PbsE were cloned into a pRSFDuet vector in a polycistronic manner, separated by ribosomal binding sequences (Table S1). Constructs were expressed in BL21 (DE3) TUNER cells, followed by immobilized metal affinity chromatography (IMAC) to purify the peptides.

First, we confirmed the production of the unmodified precursors, PbsA1-A4, by matrix-assisted laser desorption/ionization time-of-flight mass spectrometry (MALDI-TOF MS) (Figure 2D; spectra in black). Second, we coexpressed PbsA1-A4 with PbsB, PbsC, PbsD and PbsE (PbsBCDE) wherein all four precursor peptides showed mass gains of 15.99 and 31.99 Da and a mass loss of −12.02 Da with respect to the unmodified precursor (Figure 2D; spectra in brown). We focused on just one precursor (PbsA3) for subsequent experiments because of its higher solubility.

For coexpression of PbsA3 with the individual enzymes, we cloned *pbsB*, *pbsC*, *pbsD*, *pbsBE* (polycistronic), and *pbsCD* (polycistronic) into pRSFDuet vectors. Similarly, *pbsE* was cloned into pCDFDuet. PbsA3 was coexpressed with individual or different combinations of the pathway enzymes. PbsA3 did not undergo any modification (determined by a shift in mass) when coexpressed with PbsB, PbsC, PbsD or PbsE individually in (Figure 3Aii-v). However, when coexpressed with PbsCD, PbsA3 underwent mass gains of 15.99 and 31.99 Da suggesting that PbsC and PbsD acted in partnership (Figure 3Avi). PbsA3, coexpressed with PbsBCD did not undergo any further modification suggesting that the PbsCD-modified products did not serve as substrates for PbsB (Figure 3Avii). Coexpression of PbsA3 and PbsCDE resulted in a mass loss of 42 Da relative to the PbsCD-modified +31.99 Da-product, corresponding to a net mass loss of 10 Da from the unmodified peptide (Figure 3Aviii). Therefore, PbsE requires modification of PbsA3 by PbsCD for activity. We then coexpressed PbsA3 with PbsBCDE with supplementation with B_12_ (hydroxocobalamin) and use of a plasmid encoding the iron–sulfur cluster assembly proteins and the BtuCEDFB proteins for B_12_ uptake (pIGB240).^48–50^ This experiment resulted in a product that underwent a further mass loss of 2 Da relative to the PbsCDE product (Figure 3Aix and S3). Collectively, these data show that PbsB requires prior modification by PbsCD and PbsE for activity.

**Figure 3:**
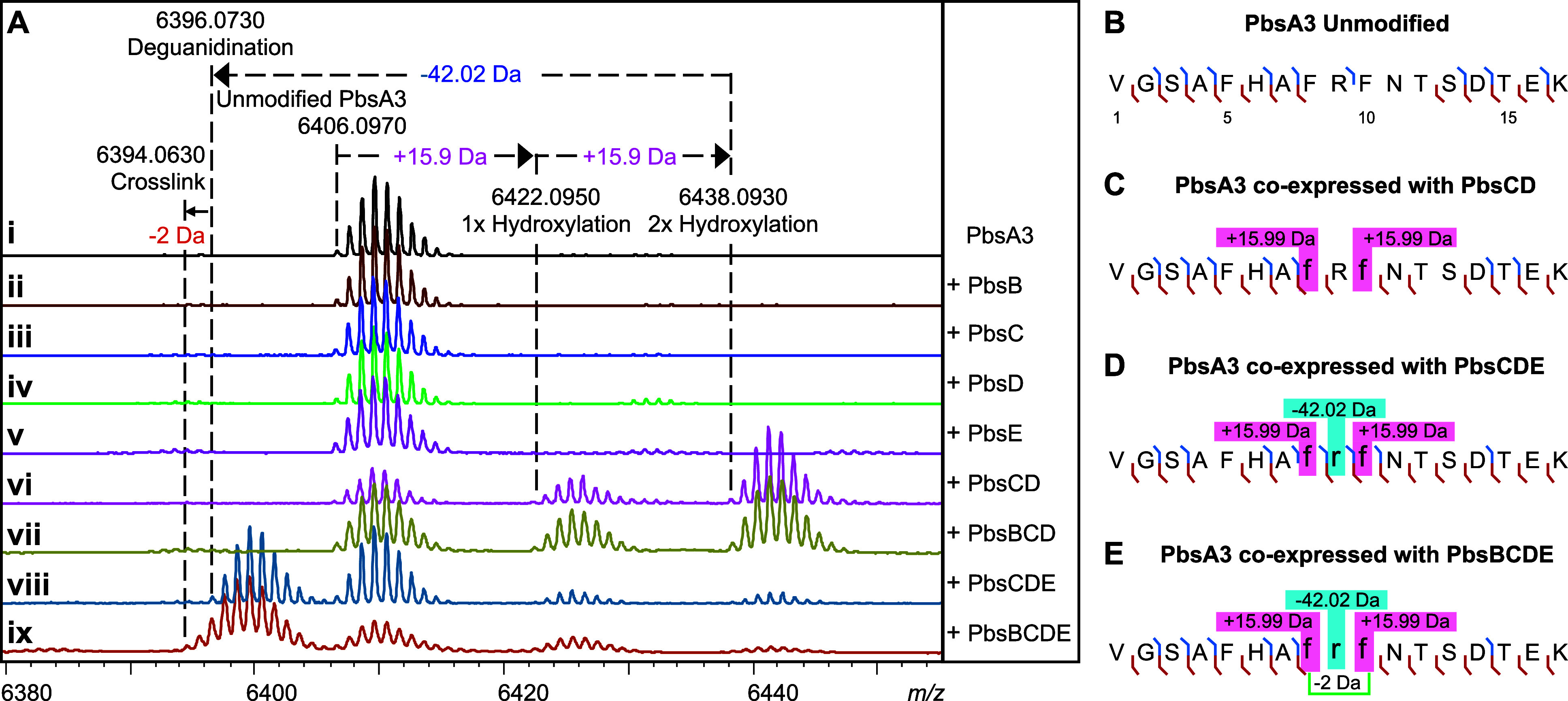
Mass spectrometric analysis of PbsA3 coexpressed with PbsB, PbsC, PbsD, and PbsE in different combinations. (A) MALDI-TOF MS spectra of the PbsA3 peptide when expressed (i) alone or coexpressed with (ii) the B12-rSAM, PbsB; (iii) the MNIO, PbsC; (iv) the RRE-containing partner protein, PbsD; (v) the arginase, PbsE; (vi) PbsC and PbsD; (vii) PbsB, PbsC, and PbsD; (viii) PbsC, PbsD, and PbsE; and (ix) PbsB, PbsC, PbsD, and PbsE. HR-MS/MS fragmentation patterns for the endopeptidase GluC-digested peptide fragments are shown for (B) unmodified PbsA3, (C) PbsA3 coexpressed with PbsCD, (D) PbsA3 coexpressed with PbsCDE, and (E) PbsA3 coexpressed with PbsBCDE. The peptide fragments after endoproteinase GluC digestion are numbered starting at Val1 (Figure 2B) with b-ions displayed in blue and y-ions displayed in red. The residues undergoing mass changes are shown in small font, further highlighted in boxes with their respective mass shifts. The hypothesized cross-linking residues are joined by a green bracket in panel E. For ESI MS/MS spectra, see Figure S4.

Through high-resolution tandem mass spectrometry (HR-MS/MS) of the GluC endopeptidase-digested PbsCD product (C-terminal fragment starting at Val1 as shown in Figure 3B), we assigned each 15.99 Da mass gain to the Phe8 and Phe10 residues (following the residue numbering in Figure 2B) in the conserved FRF motif (Figure 3C and S4B, Table S2). Similarly, the mass loss of 42.02 Da in the PbsCDE product was localized to Arg9 in the FRF-motif (Figure 3D and S4C, Table S2), suggesting that an Orn residue may be formed by the loss of a urea equivalent from the Arg side chain. For the PbsBCDE product with an additional 2 Da mass loss no fragment ions were detected inside the FRF-motif (Figure 3E and S4D, Table S2). This observation suggested the possibility of a cross-link being formed between the two modified Phe residues preventing fragmentation.

### Large-Scale Purification of Modified PbsA3

To obtain the modified core peptide of PbsA3, we cloned the putative protease PbsP into a pET28a vector with an N-terminal 6x His tag. PbsP has sequence homology with the TldDE protease family,^51–53^ but unlike this family which usually constitutes a heterodimer, the *pbs* BGC contains only a single gene (*pbsP*) and a partner protein was not identified in the genome. Attempts at purifying PbsP were unsuccessful as the desired protein was observed in inclusion bodies under various expression conditions. Therefore, endoproteinase GluC was again used to access the modified fragment peptides. PbsA3 was coexpressed with PbsCD, PbsCDE and PbsBCDE in 5 L scale and the products were purified by immobilized metal affinity chromatography (IMAC). After desalting to remove imidazole, and proteolysis by GluC endoproteinase, IMAC was performed again to remove the His-tagged leader peptide fragment and the His-tagged GluC. The collected flowthrough was subjected to HPLC purification, which led to the isolation of the bis-hydroxylated PbsA3 C-terminal fragment (which we will term PbsA3-CD henceforth; 1.2 mg/L of culture), the bis-hydroxylated and deguanidinated product, PbsA3-CDE (0.5 mg/L of culture), and the bis-hydroxylated, deguanidinated and cross-linked product, PbsA3-BCDE (0.2 mg/L of culture).

### Structural Elucidation of Modified PbsA3

The 15.99 Da mass gains on Phe8 and Phe10 in PbsA3 when coexpressed with PbsCD are typical of hydroxylation events. To probe this hypothesis, we conducted advanced Marfey’s analysis^54^ on the HPLC-purified, GluC-digested PbsA3-CD product and compared the derivatized amino acids to different hydroxylated tyrosine standards (Figure S5). Based on the LC-MS analysis, we inferred that a putative hydroxyl group was either introduced at the *ortho*-position of the Phe rings (Cδ1, Figure 4A) or on the β-carbon. We next elucidated the structures of the three products PbsA3-CD, PbsA3-CDE and PbsA3-BCDE by one-dimensional (1D) and two-dimensional (2D) NMR experiments including ^1^H–^1^H TOCSY, ^1^H–^1^H NOESY, ^1^H–^13^C HSQC and ^1^H–^13^C HMBC.

**Figure 4:**
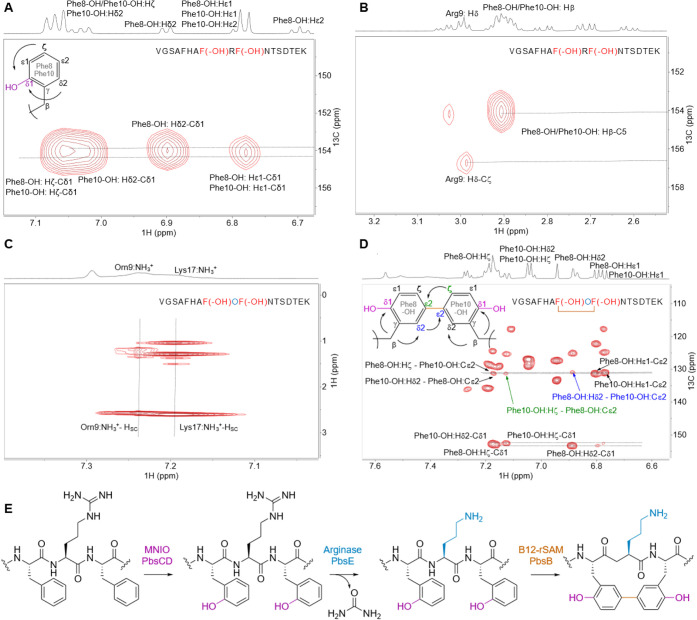
Structure determination and biosynthetic pathway of modified PbsA3 digested with endoproteinase GluC. (A) ^1^H–^13^C HMBC spectrum of PbsA3-CD highlighting the *ortho*-hydroxylated Phe8-OH and Phe10-OH residues. (B) ^1^H–^13^C HMBC spectrum highlighting the cross-peaks between the β-protons of Phe8-OH and Phe10-OH and their respective hydroxylated aromatic carbons. This part of the spectrum also shows a diagnostic cross peak for Arg9 that is consistent with an unmodified residue. (C) ^1^H–^1^H TOCSY spectrum of PbsA3-CDE collected at 3 °C in 0.1% formic acid highlighting the cross peaks of the NH_3_^+^ moiety on the Cδ of the residue at position 9 with the other side chain protons (labeled as H_sc_). (D) ^1^H–^13^C HMBC correlations in PbsA3-BCDE highlighting the cross-link formed between Cε2 carbons of the *ortho*-hydroxylated Phe8-OH and Phe10-OH residues. Cross peaks for the Hζ of Phe10-OH to the Cε2 carbon of Phe8-OH (in green arrow/font) and for the Hδ2-proton of Phe8-OH to the Cε2 carbon of Phe10-OH (in blue arrow/font) are shown. For panels A–D, the sequences of the analyzed peptides are shown in the spectra. Modified residues are in red (hydroxylated Phe8/10) or blue font (ornithine). (E) Biosynthetic pathway of PbsA3 modified by PbsCD, followed by PbsE and PbsB.

For the PbsA3-CD peptide, all backbone and side chain protons including the 16 amide protons of the 17-mer peptide were observed in a TOCSY spectrum of the sample in 90% H_2_O and 10% D_2_O (Table S3). The aromatic protons of Phe8 and Phe10 showed different splitting patterns from a normal Phe residue. Their aromatic side chain protons integrated to four protons, and the 1D ^1^H–^1^H TOCSY spectra showed four protons that displayed two doublets and two triplets (Figure S6A). More detailed analysis of ^1^H–^13^C HSQC and HMBC spectra was consistent with the addition of a hydroxyl (OH) group at the *ortho*-position (Cδ1, Figure 4A). Both β-protons of Phe8 and Phe10 showed a cross peak to their corresponding Cδ1 carbon (C5) at 154.1 ppm (Figure 4B). This assignment is consistent with the 15.998 and 31.998 Da mass gains for the PbsA3-CD product that were observed by HR-MS/MS and indicated that both Phe residues in the conserved FRF-motif where hydroxylated to produce *ortho*-Tyr.

In the advanced Marfey’s analysis the hydroxylated Phe residues coeluted with both the l-*ortho*-tyrosine and dl-3-hydroxy-phenylserine standards. However, hydroxylation of Cβ is inconsistent with the observed integration in the NMR spectra indicating four protons at each of the aromatic rings of Phe8 and Phe10 (Figure S6A). Additionally, the ^1^H–^13^C multiplicity-edited HSQC showed the Cβ as a methylene group and not a methine (Figure S6B). Thus, both Phe residues were hydroxylated at Cδ1.

To confirm the formation of Orn by PbsE and that no isobaric modification (e.g., epimerization) was performed on this residue by the rSAM PbsB, we conducted advanced Marfey’s analysis on the GluC-digested and HPLC-purified PbsA3-CDE and PbsA3-BCDE peptides (Figure S7). LC-MS analysis of the derivatized samples and l-Orn, d-Orn and l-Arg standards supported the conclusion that the Arg was converted into l-Orn by PbsE and that no epimerization had occurred.

Formation of Orn was confirmed by NMR spectroscopic analysis. The ^1^H spectrum of the PbsA3-CDE product was similar to that of PbsA3-CD (Figure S8). The 1D ^1^H–^1^H TOCSY showed four protons each for the aromatic side chains of Phe8 and Phe10 that were also observed in the PbsA3-CD product (Figure S9). The major difference between PbsA3-CDE and PbsA3-CD was the disappearance of the side chain NHε proton of Arg9 in the PbsA3-CDE spectrum (Figure S8B). In PbsA3-CD, the NHε peak formed a sharp triplet at 6.99 ppm showing cross peaks with the other side chain protons and the amide proton of Arg9 in the TOCSY spectrum (Figure S10). However, in PbsA3-CDE, this peak was not detected (Figure S8B and Table S4). After lowering the temperature to 3 °C to slow down exchange with bulk water and adding 0.1% formic acid to maintain the NHε in a protonated state, a broad peak was observed at 7.46 ppm integrating for approximately three protons, which showed cross peaks in a TOCSY spectrum with the other side chain protons of the residue at the ninth position in the peptide, consistent with a protonated primary amine group (Figure 4C and S11). All of these observations provided further evidence that Arg9 was transformed into an Orn residue.

For PbsA3-BCDE all backbone and side chain protons including 16 amide protons were observed in a TOCSY spectrum of a sample in 90% H_2_O and 10% D_2_O with 0.1% formic acid-d_2_ (^1^H and ^13^C NMR assignments in Table S5). Compared to the PbsA3-CD and PbsA3-CDE peptides, the aromatic protons of Phe8-OH and Phe10-OH in PbsA3-BCDE showed splitting patterns unlike the aromatic side chain of Phe and also different from the aromatic peaks observed in the PbsA3-CD and PbsA3-CDE peptides. In PbsA3-BCDE, integration of the aromatic signals from the former Phe8-OH and Phe10-OH indicated three protons each, and the 1D TOCSY spectrum (Figure S12) showed for each residue one doublet (d, *J* = 8.5 Hz), one doublet of doublets (dd, *J* = 8.5 Hz, 1.9 Hz) and one singlet (br). The ^1^H–^13^C HMBC revealed a C–C cross-link between the Cε2-carbons of Phe8-OH and Phe10-OH (Figure 4D,E). The β-protons of both Phe8-OH and Phe10-OH showed cross peaks to the hydroxylated Cδ1 carbons, as well as to the Cδ2-carbons (Figure S13). Additionally, the presence of a cross-link between the two Cε2-carbons of the aromatic rings of Phe8-OH and Phe10-OH was suggested by the observation of additional cross peaks between the two rings. For example, the peak at 7.12 ppm (Hξ of Phe10-OH) showed a cross peak to the Cε2 carbon of Phe8-OH at 131.3 ppm (Figure 4D, green arrow). Similarly, the Hδ2-proton at 6.89 ppm of Phe8-OH showed a cross peak to the Cε2 carbon of Phe10-OH at 131.0 ppm (Figure 4D, blue arrow). The HMBC bond connectivity of Phe8-OH and Phe10-OH in the PbsA3-BCDE peptide is depicted in Figure 4D. The NMR analysis also confirmed an Orn instead of an Arg at position 9, based on the same analysis as discussed above for the PbsA3-CDE peptide (Figure S14).

### Investigation of Unmodified Residues in the Conserved FHAFRF Motif

All enzymatic modifications are restricted to the FRF region of PbsA3, but the sequence that is fully conserved in all precursor peptides is considerably larger (AFHAFRF, Figure 2C). Therefore, we investigated the importance of the conserved Phe5 and His6 residues by replacement with Ala (F5A and H6A; residue numbering from Figure 2B) and coexpression with PbsCD and PbsBCDE in . The F5A variant resulted in significantly reduced conversion by the MNIO (Figure S15A; red spectra). No arginase- and B12-rSAM enzyme-mediated modification was observed, likely because of the low hydroxylation activity, which is required for PbsB and PbsE activity (Figure S15A; orange spectra). The MNIO retained considerable activity with the H6A variant, resulting in wild type-like bis-hydroxylation (Figure S15B; red spectra), but the arginase and the rSAM enzymes did not introduce any further modifications (Figure S15B; orange spectra). Together, these results suggest that Phe5 is important for MNIO catalysis and that His6 is not necessary for MNIO activity but seems critical for the activity of the arginase PbsE and the B12-rSAM PbsB.

### *In Vitro* Reconstitution Of Enzyme Activity

Following the reconstitution of the PbsA3 biosynthetic pathway in , we focused on *in vitro* reconstitution of the individual enzyme activities. For the purification of PbsCD, we constructed a pRSFDuet plasmid encoding an N-terminally 6xHis-tagged PbsC. The plasmid also contained untagged *pbsD* under a second T7 promoter in the multiple cloning site (MCS) 2 of the pRSFDuet vector. When expressed in , PbsD copurified with PbsC, validating that PbsD is an interacting partner for PbsC (Figure S16). HPLC-purified unmodified PbsA3 was reacted with the as-purified PbsCD, and no modification was observed (Figure 5Aii). Addition of freshly prepared FeSO_4_ to the reaction (under aerobic conditions) led to the formation of mono- and bis-hydroxylated products (Figure 5Aiii), suggesting the involvement of Fe­(II) ions in enzyme catalysis. Preincubation of as-purified PbsCD with sodium ascorbate (for possible reduction of enzyme-bound Fe­(III) to Fe­(II) in the enzyme active site) followed by addition of PbsA3 also led to the formation of mono- and bis-hydroxylated products (Figure 5Aiv).

**Figure 5:**
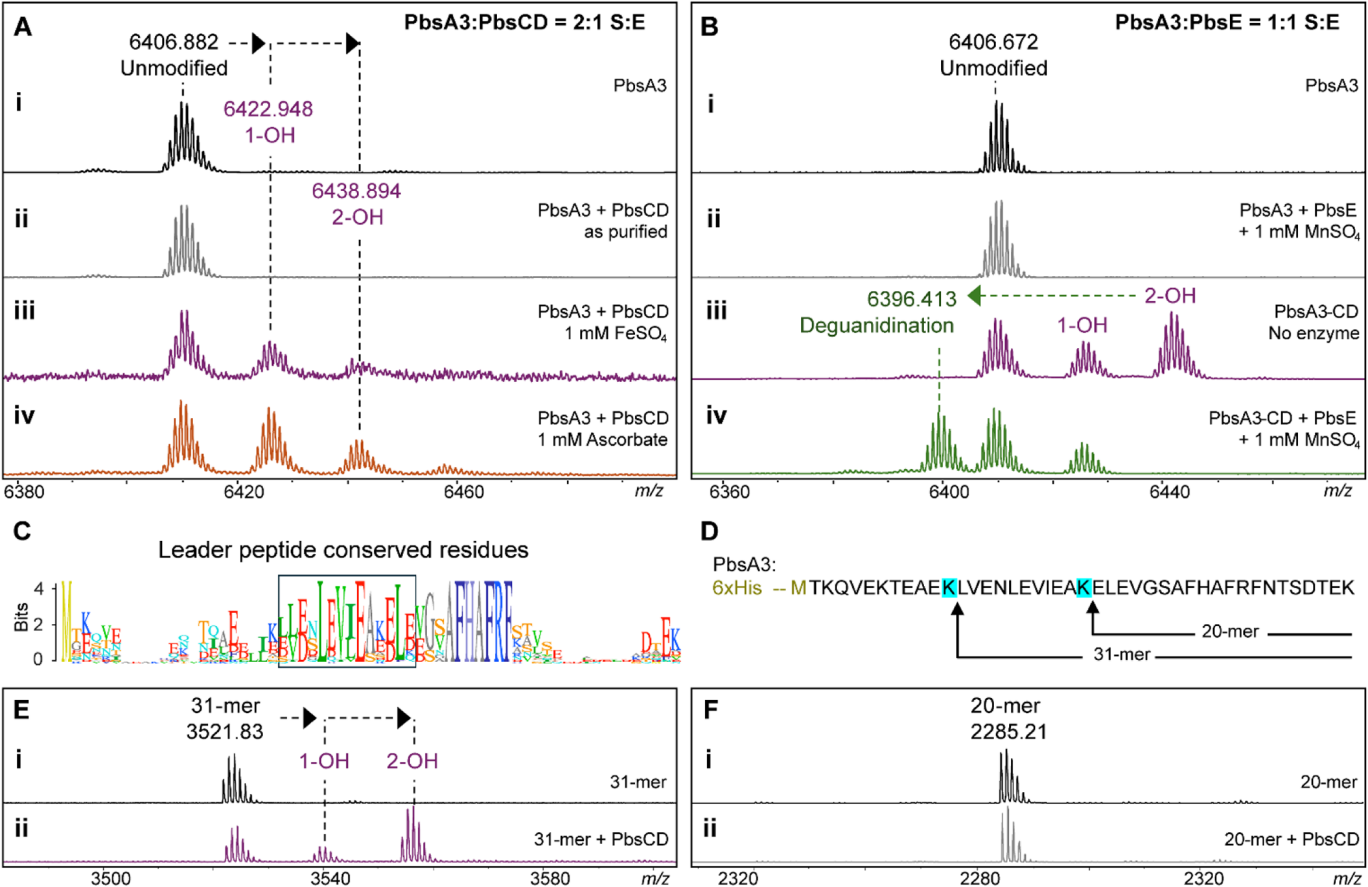
In vitro reconstitution of MNIO and arginase activity and minimal substrate determination for the MNIO. (A) MALDI-TOF MS spectra of PbsA3 after in vitro reaction with (i) no enzyme, (ii) PbsCD, as isolated; (iii) PbsCD, in the presence of FeSO_4_; and (iv) PbsCD, in the presence of sodium ascorbate. (B) MALDI-TOF mass spectra of PbsA3 after in vitro reaction with (i) no enzyme and (ii) PbsE in the presence of MnSO_4_. No arginase activity was detected on unmodified PbsA3. MALDI-TOF mass spectra of (iii) PbsCD-modified PbsA3, containing a mixture of unmodified, singly hydroxylated and bis-hydroxylated product, and (iv) PbsA3-CD when reacted in vitro with PbsE in the presence of MnSO_4_. (C) Sequence logo of the FHAFRF motif containing precursors showing conserved residues in the leader peptide, highlighted in the box. (D) Partial LysC proteolysis of PbsA3 generated 20-mer and 31-mer fragments that were purified by HPLC. The 31-mer fragment contains the conserved residues of the leader peptide, which are absent in the 20-mer fragment. (E) MALDI-TOF mass spectra of the 31-mer fragment after in vitro reaction with (i) no enzyme and (ii) PbsCD in the presence of sodium ascorbate. (F) MALDI-TOF mass spectra of the 20-mer fragment after in vitro reaction with (i) no enzyme and (ii) PbsCD in the presence of sodium ascorbate.

For the purification of PbsE, another plasmid was constructed to express the enzyme with an N-terminal 6xHis tag. The purified 6xHis-TEV-PbsE fusion protein was directly used for *in vitro* assays (in the presence of manganese sulfate) with either unmodified PbsA3 (Figure 5Bii), or the PbsCD product containing PbsA3 and mono- and bis-hydroxylated PbsA3 (Figure 5Biii, iv). As observed in the *in vivo* coexpression studies, PbsE selectively modified the bis-hydroxylated product whereas unmodified PbsA3 and the monohydroxylated product did not undergo deguanidination (Figure 5Biv). Apart from establishing the *in vitro* activity of PbsE, this observation validated our hypothesis that the PbsCD-mediated hydroxylation is the first step in the biosynthetic pathway, providing the substrate for the arginase PbsE. Attempts to reconstitute the activity of PbsB in vitro have not been successful.

### Minimal Substrate Required for MNIO Activity

Sequence alignment of the predicted precursors containing the conserved core motifs also revealed a conservation pattern in the putative leader peptide (LP) region (Figure 5C). To investigate the importance of these residues, we made truncated substrates by conducting a partial digestion of the unmodified PbsA3 peptide using LysC endopeptidase. After 15 min at room temperature with 1:10,000 enzyme:substrate, peptide fragments of varying lengths were isolated by HPLC (Figure 5D) including a 20-mer fragment without the conserved LP stretch, and a 31-mer fragment with the conserved stretch of amino acids in the LP (Figure 5D). These purified fragments were reacted with PbsCD *in vitro*, which resulted in the successful bis-hydroxylation of the 31-mer fragment (Figure 5E). No modification was observed for the 20-mer fragment, suggesting that the conserved LP region is important for MNIO activity (Figure 5F).

### Determining the Substrate Scope of PbsCD

To investigate the possibility of a required order of hydroxylation of Phe8 and Phe10, we mutated these residues to Ala. When coexpressed with PbsCD or PbsBCDE in , the F8A and F10A variants (hereafter named **A**RF and FR**A**-variants, respectively, mutated residues in bold) underwent only one hydroxylation (Figure S17A,B). Using HR-MS/MS we assigned these hydroxylations to the Phe residues (Figures S18 and S19). Upon coexpression with PbsBCDE, no arginase activity and no PbsB-mediated cross-linking was detected for either peptide suggesting that both Phe8 and Phe10 need to be hydroxylated for these activities. As expected based on the data in the previous sections, the ARA variant of PbsA3 was not modified by any of the enzymes (Figure S17C).

The SSN cluster harboring PbsC (Figure 2A) also contains enzymes that are encoded in BGCs with precursor peptides containing alternative conserved motifs in the core region, such as FHTFMF and YHxYxY motifs (Figure S1). We therefore made a series of variants of PbsA3 to investigate if the Pbs enzymes would accept these as substrates. The **Y**RF, FR**Y** and **Y**R**Y** variants were processed by PbsCD (Figure S20) resulting in mostly one and two hydroxylations (Figures S21–S23). The patterns observed in HR-MS/MS analysis showed the hydroxylation to occur on the aromatic residues. The fragment ions for the doubly hydroxylated **Y**R**Y** product suggested that both Tyr8 and Tyr10 were modified (Figure S23). Coexpression of the **Y**RF, FR**Y** and **Y**R**Y** variants with PbsBCDE resulted in successful arginase activity for all three variants (Figure S20). Only the **Y**RF and FR**Y** variants were accepted as substrates for PbsB resulting in a cross-link as inferred from the 2 Da mass loss in HR-MS. These data show that PbsCD can hydroxylate Tyr residues, consistent with the motifs seen in the substrates for PbsCD orthologs.

### Characterization of Tyrosine Modifications Catalyzed by Pbs Enzymes

PbsA3-YRY variants were bis-hydroxylated by PbsCD, but not cross-linked by PbsB (Figure S20C). We therefore replaced the FRF motif in PbsA3 with the YTY motif found in the precursor from the *stg* BGC (Figure S2) in the genome of ATCC 700248 (YHTYTY-motif). Upon coexpression with PbsCD, the PbsA3-YTY precursor was successfully bis-hydroxylated (YTY-CD product; Figures S24B and S25B; Table S1). A monohydroxylated product (YTY-CD 1-OH; Figure S24B and Table S1) was also detected with the hydroxylation localized on Tyr10 as determined by HR-MS/MS (Figure S25A). Upon coexpression with PbsBCD, besides the mono- and bis-hydroxylated products, we also observed cross-linking of the PbsA3-YTY precursor that was not hydroxylated (YTY-B product; Figures S24B and S26A) showing that PbsB can macrocyclize tyrosines independent of MNIO-mediated hydroxylation. In addition to the aforementioned products formed in the PbsBCD coexpression, we also observed a minor bis-hydroxylated and cross-linked product (YTY-BCD 2-OH) along with a minor monohydroxylated-cross-linked product (YTY-BCD 1-OH; Figures S24B and S26B and Table S1).

### Structure Elucidation of YTY-B and the Two Products of YTY-CD

Large-scale purification of the PbsA3-YTY products from coexpression with PbsCD (YTY-CD) and PbsB (YTY-B) was conducted followed by GluC endoproteinase digestion and subsequent HPLC purification. The NMR structures of the YTY-B and mono and bishydroxylated YTY-CD peptides were determined by 1D and 2D NMR experiments (^1^H–^1^H TOCSY, ^1^H–^1^H NOESY, ^1^H–^13^C HSQC, and ^1^H–^13^C HMBC). For the YTY-B peptide, all backbone and side-chain protons including 16 amide protons of the 17-mer peptide were observed in the TOCSY spectrum for the sample in 90% H_2_O and 10% D_2_O and 0.1% formic acid-d_2_ (Table S6). The aromatic protons of Tyr8 and Tyr10 showed different splitting patterns from a normal Tyr, integrating to three protons each. The 1D TOCSY spectrum showed three protons associated with two doublet peaks and 1 singlet (or 1 doublet with a small *J* coupling of 2.5 Hz; Figure S27). Detailed analysis of ^1^H–^13^C HSQC (Figure S28) and HMBC (Figure S29) data revealed a C–C cross-link between the two aromatic rings of Tyr8 and Tyr10 at the Cε2 positions. The Hδ2 proton at 6.64 ppm of Tyr8 showed a cross peak to the Cε2 carbon at 125.4 ppm of Tyr10. Both beta protons of Tyr8 and Tyr10 showed NOE cross-peaks to their respective Hδ1 and Hδ2 protons in a ^1^H–^1^H NOESY spectrum, which provides additional support that the C–C cross-link occurred between the Cε2 carbons of the two rings (Figure S30). The structure in Figure 6 is also consistent with the HR-MS and HR-MS/MS analysis as shown in Figures S24B and S26A, respectively.

**Figure 6:**
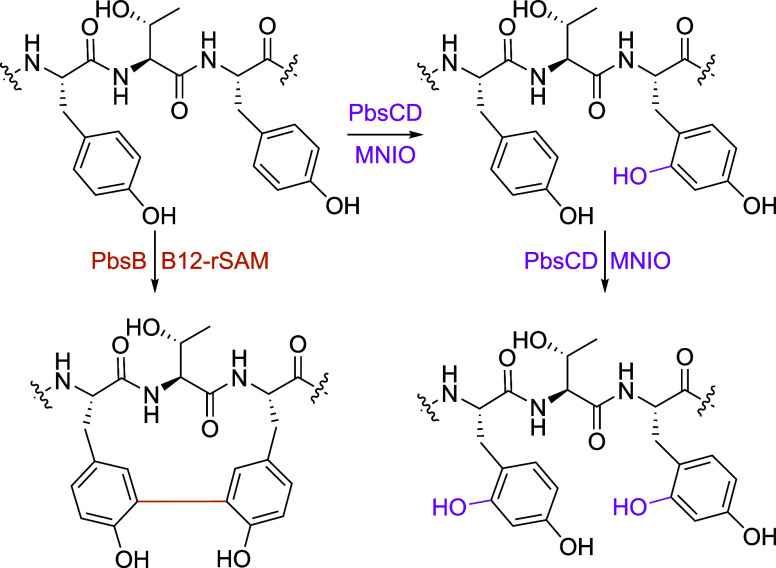
PbsA3-YTY variant modification catalyzed by PbsBCD. The B12-rSAM enzyme PbsB-mediated cross-linking of Tyr residues in the PbsA3-YTY variant. PbsCD first hydroxylates the Tyr10 residue and then Tyr8.

The mono and bishydroxylated YTY-CD peptides coeluted during HPLC purification. Hence, their structural characterization was conducted as a mixture. Although the ^1^H spectrum is complicated, two sets of signals with a ratio of 2:3 (based on peak integration values) were clearly observed. The 1D ^1^H–^1^H TOCSY spectrum in the aromatic region revealed two distinct types of spin systems (Figure S31). One displayed the original pattern of a Tyr residue, the other showed three peaks for each ring: one doublet, one doublet of doublets, and one singlet. Based on the proton integration values, one of the peptides contained one intact Tyr residue and one modified Tyr residue, whereas the other contained two modified Tyr residues. Further analysis of 2D ^1^H–^1^H TOCSY (Figure S32), ^1^H–^13^C HSQC (Figure S33) and HMBC (Figure S34) revealed that the modified Tyr residue was hydroxylated at the Cδ1 position. The proton Hδ2 at 6.80 ppm of Tyr8 in the bishydroxylated YTY-CD peptide showed a cross peak to the Cδ1 carbon at 155.3 ppm and a cross peak to the Cζ carbon at 155.7 ppm (carrying the original −OH of Tyr). Likewise, the Hδ2 proton at 6.87 ppm of Tyr10 in monohydroxylated YTY-CD showed a cross peak to the Cδ1 carbon at 155.3 ppm and a cross peak to its original Cζ–OH at 155.7 ppm (Figure S34). The beta-protons of Tyr10 in this peptide showed cross peaks to their respective downfield shifted Cδ1 carbon at 155.3 ppm (Figure S35), confirming the location of the hydroxylation. The assignments for both peptides are given in Table S7, and the structures of both peptides are consistent with the HR-MS/MS results (Figure S25). Collectively, these data show that the site of hydroxylation by PbsCD is the same for Phe and Tyr, and that PbsB cross-links Tyr, *ortho-*Tyr and Cδ1-hydroxy-Tyr residues with the same regiochemistry.

### AlphaFold 3 Prediction of Pbs Pathway Enzymes in Complex with Precursor Peptides

To understand the mechanism of substrate recognition and modification by PbsCD, we generated AlphaFold 3 models^55^ of PbsCD bound to PbsA1-A4 and three iron ions (Figure S36; model shown for PbsCD in complex with PbsA2). The predicted model showed PbsC and PbsD as heterodimeric molecules, consistent with copurification of untagged PbsD with tagged PbsC (Figure S16). The per-residue measure of local confidence (pLDDT) values for both proteins are generally very high (>90), with lower confidence in the orientation of the PbsA substrate peptides. The model suggests an antiparallel β-sheet interaction between a conserved region of the leader peptide of the four precursors (Figures S36B and S37) with the RiPP recognition element (RRE) in the PbsD partner protein, as observed in various other enzymes for which the substrate engagement has been structurally characterized.^56–63^ The model placed three Fe ions in the putative active site of the enzyme (Figure S36C), consistent with other MNIO structures, although it is still unclear whether the active form of this family of enzymes contains two or three Fe ions.^23^ His97, Asp133 and Glu178 are predicted to coordinate with one Fe ion, His216, Asp260 and His262 with a second Fe ion, and Asp213, His247 and Glu290 with the third iron. Interestingly, for PbsA1, A2 and A4, although the pLDDT values are considerably lower, the predicted substrate-bound structure placed one of the two Phe residues in the FRF motif in close proximity to the Fe ions in the enzyme active site (Figure S38A,B and D). However, for the PbsA3-complexed model, the His residue (His5) of the FHAFRF motif was placed in proximity to the active site metals (Figure S38C). Since PbsA3 has been experimentally shown to undergo modification at Phe8/Phe10 residues, the prediction shows the limitations of AlphaFold 3 and the need for experimental validations of the model. Nevertheless, the predicted complexes shed light on the putative RRE-containing partner protein that may interact with the leader region, helping the enzyme recognize the substrate.

A similar prediction was also made for the arginase PbsE in conjunction with the precursors PbsA1-A4 and two manganese ions (Figure S39A). The putative metal-coordinating residues in the active site were predicted to be His10, Asp31, His33, Asp35, Asp165 and Asp167 (Figure S39B). The model for the substrate-bound complex placed Arg9 residue of the FHAFRF motif proximal to the Mn ions (Figure S39D–G). In general, we believe that the complexes of the arginase and the unmodified precursor predictions may not be completely reliable, considering the specific selectivity of PbsE for PbsCD-modified bis-hydroxylated intermediates as the substrate. We did not use AlphaFold to model the B12-rSAM PbsB and the unmodified precursor interactions since the enzyme selectively cross-links bis-hydroxylated and deguanidinated intermediates, which are not accessible in the current versions of AlphaFold3.

### PbsA3-BCDE Product Shares Structural Similarity with Biphenomycins

The unusual cross-linked peptide structure assembled by PbsBCDE is reminiscent of the core structure common to the biphenomycin antibiotics produced by NRRL 3217 ^34,35^ (also called LL-AF283) and No. 43708 ^36,37,64^ (also called WS-43708A), with a few key differences (Figure 7A). All biphenomycin analogs contain a hydroxyl group at the γ-carbon of the Orn residue, while biphenomycin A and C also harbor a β-hydroxylation at one of the *ortho*-tyrosine residues in the macrocycle. No information on biphenomycin biosynthesis has been reported. We thus sequenced the genome of NRRL 3217, which enabled identification of a BGC (*bpm* cluster; Figure 7B, NCBI Accession ID: PV659837) that is very similar to the *pbs* BGC. The *bpm* cluster encodes a putative precursor peptide (BpmA) that contains the conserved AFHAFRF motif followed by an Arg and a Ser. In addition, genes predicted to encode an arginase (BpmC), a B12-rSAM (BpmD), an MNIO (BpmE), and a RiPP recognition element-containing protein (BpmF) were identified in the BGC (Figure 7B and Table S8). We propose that these enzymes perform the same chemistry as their Pbs orthologs on the FRF motif in BpmA. Three additional genes are predicted to encode a JmjC domain-containing hydroxylase (BpmG), an α-ketoglutarate HExxH-type peptide β-hydroxylase (BpmH), and a TldD-type metallopeptidase (BpmI). These enzymes are likely responsible for the two additional side-chain hydroxylations and proteolytic trimming to arrive at biphenomycin A.^39^ These findings are fully consistent with the *bpm* BGC directing biphenomycin biosynthesis in NRRL 3217.

**Figure 7:**
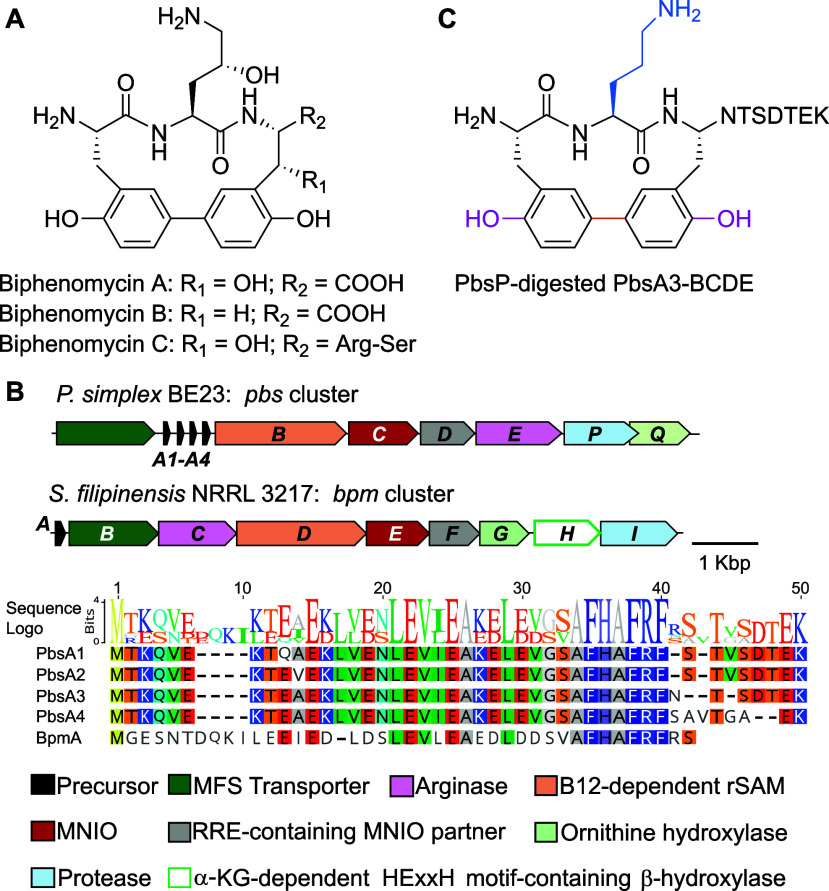
Biphenomycin structure and its BGC. (A) Structures of biphenomycins A, B, and C. (B) BGC identified in one of the biphenomycin producer strains, NRRL 3217, encoding enzymes that are homologous to the Pbs enzymes characterized in this study. The corresponding precursors are aligned. (C) Structure of PbsP-digested PbsA3-BCDE displaying structural similarity with the biphenomycins.

Comparison of the *pbs* and *bpm* gene clusters revealed the presence of *pbsQ*, encoding a cupin-like domain-containing protein related to the JmjC domain-containing protein BpmG, in the *pbs* BGC (Figure 7B). The start codon for *pbsQ* lies several base pairs upstream of the *pbsP* stop codon*.* Coexpression of the Pbs A1-A4 precursors with recombinant PbsQ and PbsCD did not yield any further modification of the MNIO-modified bis-hydroxylated products (Figures S40–S43). However, coexpression of the precursors with PbsQ and PbsCDE (i.e., inclusion of the arginase) resulted in an additional 15.99 Da mass gain equivalent to a hydroxyl group (Figures S40–S43), which was localized to the Orn9 residue by HR-MS/MS (Figure S44). This finding suggest that PbsQ selectively hydroxylates the Orn residue and acts after PbsCDE. Because of the low conversion and formation of a mixture of products, further structural characterization of the PbsCDEQ products was not performed. Unlike the biphenomycin BGC, a gene encoding for a BpmH homologue was not present in the *pbs* BGC.

In order to directly connect the *pbs* pathway products to the *bpm* cluster, we also coexpressed the Pbs precursors with PbsCDE and the Orn hydroxylase (BpmG) from the *bpm* BGC. All four precursors PbsA1-A4 underwent an additional 15.99 Da mass gain in addition to the bis-hydroxylation and deguadinination catalyzed by the MNIO and the arginase, respectively. Similar to the modification by PbsQ, BpmG-mediated hydroxylation was localized to the Orn9 residue in PbsA1-A4 by HR-MS/MS analysis (Figures S45 and S46). These findings convincingly link the *bpm* pathway to the Pbs pathway and in turn the *pbs* pathway to the biphenomycins.

### Characterization of the Protease PbsP

Prompted by the link between the biphenomycins and the product of the *pbs* cluster, we returned to the PbsP protease and added an N-terminal maltose binding protein (MBP) solubility tag to the previously insoluble enzyme. The MBP-PbsP fusion protein was purified and successfully processed all four PbsBCDE-modified precursor peptides (Figure S47) in the presence of ZnSO_4_ as determined by HR-MS/MS analysis (Figure S48). We did not observe any C-terminal proteolysis. We believe the homologous protease encoded in the *bpm* cluster would act in a similar manner to form biphenomycin C. Further maturation of biphenomycin C to its A and B forms may be catalyzed by a generic carboxypeptidase encoded elsewhere in the genome of the producer.

## Discussion

Enzymes involved in posttranslational modifications (PTMs) of RiPPs offer a plethora of diverse and interesting chemical transformations that are challenging to perform synthetically.^20^ To identify enzymes with new functions, SSNs are a valuable tool to group thousands of uncharacterized enzymes based on variable sequence similarity cut-offs. In many cases, enzymes that make up one cluster have been reported as being isofunctional.^65,66^ In this study, we made an SSN for MNIOs and focused on associated substrate peptides containing conserved motifs that were predicted to result in novel chemistry. Indeed, investigation of one such BGC uncovered five different metalloenzymes involved in the PTM of a conserved FHAFRF motif-containing RiPP precursor from the genome of BE23.

Prior to this study, MNIO-catalyzed reactions had been characterized that involve modification of Cys, Asn, and Asp residues (Figure 1);^23^ aromatic residues had not been reported as sites for MNIO modification. The hydroxylation of aromatic side chains reported here thereby further expands the diverse range of chemical reactions performed by MNIOs. Hydroxylation of aromatic amino acids has been reported to be catalyzed by heme-containing cytochrome P450 enzymes, pterin-dependent hydroxylases, flavin-dependent monooxygenases, nonheme mononuclear iron dioxygenases and diiron hydroxylases.^67–70^ While enzymatic *ortho*-hydroxylation of phenolic moieties has been reported for certain natural products,^71^ reports on Cδ1-hydroxylation of Phe residues in RiPPs are scarce. To the best of our knowledge, the only known example is in the case of thioviridamide-like compounds where *ortho*-hydroxylation of a Phe residue is catalyzed by a cytochrome P450 enzyme.^72^

AlphaFold3 prediction models of the MNIO and precursor substrate complexes provide structural insights of the interaction of the MNIO and precursor peptides. The RRE-containing partner protein PbsD likely recognizes the substrate through an antiparallel β-sheet interaction with the leader region of the precursor peptide. Consistent with the model, deletion of the leader fragment predicted to form the beta-sheet led to loss of enzymatic processing (Figure 5F). Interestingly, in all the *pbs* precursors except PbsA3, one of the Phe residues involved in modification was placed close to the Fe ions in the predicted active site of the MNIO in the AlphaFold3 models. The mechanism of hydroxylation of the two Phe residues in the FRF motif is at present not clear. Most MNIOs catalyze four-electron oxidations of their substrates, shuttling the four electron equivalents to molecular oxygen and regenerating a ferrous ion that can initiate the next catalytic cycle.^23^ Only one MNIO has thus far been shown to catalyze a two-electron oxidation, MovX that oxidatively cleaves the Cα−N bond in an Asn residue generating hydrogen peroxide in the process (Figure 1).^28^ Furthermore, thus far, all proposed MNIO mechanisms have involved C–H activation at an sp^3^–hybridized carbon, which has been hypothesized to be carried out by a ferric superoxide species.^23^ The double hydroxylation of two Phe residues in PbsA in principle could be a four-electron process in which both oxygens from O_2_ are incorporated into two different Phe residues, but that would require the oxidations of the two Phe residues to be carried out by two different oxidating species on PbsC (e.g., Figure S49). Alternatively, the reaction could involve two sequential two-electron oxidations that each involve a different molecule of O_2_. In other enzymes that carry out aromatic ring oxidations such as cytochrome P450 or tetrahydrobiopterin-dependent enzymes, an Fe­(IV)-oxo is required.^69,70^ If PbsC uses an Fe­(IV)-oxo or a peroxo species for the two-electron oxidation of one of the Phe residues (Figure 8A,B), reducing equivalents would be needed from the environment. The observation that ascorbate activated the enzyme (Figure 5Aiv) could be consistent with such a mechanism, but detailed studies will be needed to distinguish the various possibilities.

**Figure 8:**
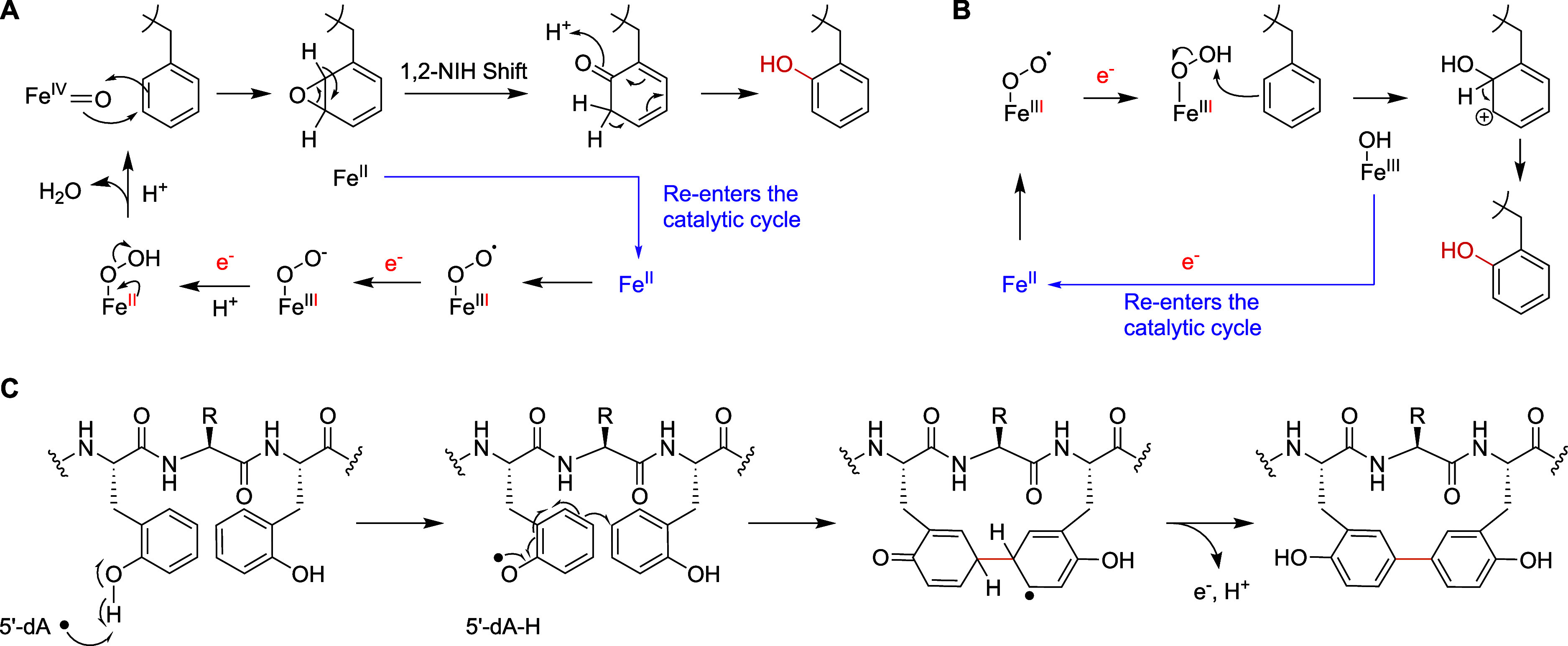
Possible reaction mechanisms for the MNIO and B12-dependent rSAM enzyme from the *pbs* BGC. (A) Possible mechanism of hydroxylation catalyzed by the MNIO PbsC using an Fe­(IV)-oxo through an epoxide intermediate followed by a hydride shift (1,2-NIH shift).^73^ Other mechanisms that do not involve a hydride migration (electrophilic aromatic substitution like) can also be drawn. (B) An alternate mechanism potentially initiated by a Fe­(III)-peroxo species. (C) Proposed mechanism of C–C cross-linking between the hydroxylated Phe8 and Phe10 residues initiated by hydrogen abstraction by a 5′-deoxyadenosyl radical.

Ornithine-containing peptides are found in both nonribosomal peptides and RiPPs. Orn is present in various bioactive compounds including the biphenomycins^37^ and landornamide.^74^ In the latter case, two ornithine residues are installed by the arginase OspR, which showed considerable substrate tolerance.^75^ In contrast, the arginase PbsE selectively acts on an Arg residue that is bordered by hydroxylated Phe residues. Such selectivity perhaps guides the directionality of the *pbs* biosynthetic pathway and provides the substrate for downstream processing by the Orn hydroxylase and the B12-dependent rSAM enzyme. Interestingly, orthologous precursor peptides missing the conserved Arg residue also lacked the gene for an arginase in the BGC suggesting a precursor-mediated evolution of the BGCs across different organisms. Similarly, BGCs containing substrates that do not contain the conserved Arg residue also lack a gene for a PbsQ homologue that hydroxylates the Orn. Like PbsE, PbsQ acts in a strictly ordered manner after Phe hydroxylation and Orn formation whereas Arg was not hydroxylated.

Aromatic side-chain cross-linking in RiPP biosynthesis is well-studied.^76^ C–C cross-link formation between the aromatic side chains of Tyr has been reported for cytochrome P450 enzymes involved in the biosynthesis of cittilin,^77^ arylomycin,^78^ the biarylitides,^79–82^ pyruvatides,^29^ and vancomycin,^83,84^ but has not been reported for B12-rSAMs. Other families of rSAM enzymes generate cross-links between the side chains of various aliphatic amino acids and the side chains of aromatic amino acids in the triceptide group of RiPPs.^85,86^ The mechanism of these latter reactions involves hydrogen atom abstraction from the aliphatic amino acid and then addition of the resulting carbon-based radical to the aromatic rings of the partner amino acid.^83,87^ PbsB on the other hand cross-links two aromatic amino acids, a process that has not been previously reported for a B12-rSAM enzyme. Originally believed to be limited to methylation,^88^ this group of enzymes has been implicated in a variety of other transformations including ring rearrangement reactions.^89^ Regarding the possible mechanism of the PbsB-catalyzed cross-linking reaction, the required MNIO-catalyzed introduction of hydroxyl groups on two Phe residues likely activates the aromatic rings, by making the aromatic ring more electron rich and potentially by providing a site for hydrogen atom abstraction from the phenol. We suggest the formation of an *ortho*-Tyr radical by hydrogen atom transfer to the canonical 5′-deoxyadenosyl radical (Figure 8C), which results in spin density at the Cε2 carbon. Addition to the partner *ortho-*Tyr in the confines of the enzyme active site would result in an aromatic radical that can be oxidized by one electron (possibly to an FeS cluster as shown for streptide)^87^ and be deprotonated to rearomatize the ring (tautomerization also rearomatizes the other ring). It is not clear, however, why the reaction requires a vitamin B12-dependent rSAM enzyme as the proposed mechanism does not suggest an obvious role for cobalamin. It is possible that the B12-dependence is vestigial or that it acts as an electron transfer conduit as suggested for the ring contraction B12-rSAM enzyme OxsB.^90^ Our data show that the YTY sequence gave rise to the same cross-linking pattern, consistent with the hypothesis that cyclization requires phenolic substrates. The position of hydroxylation (on Cδ1 or on Cζ) appears not critical for cyclization as the resulting tyrosyl radicals are both activated for coupling at Cε2. But if the mechanism in Figure 8C is indeed operational, then it is surprising that the 5′-deoxy adenosyl radical would be able to perform hydrogen atom abstraction from a phenolic O–H bond at the δ1 position as well as the ζ position. Another interesting question is why MNIO activation of Phe residues would have evolved when Tyr residues apparently give the same cyclized framework. One possibility is that cross-linking of two *ortho-*Tyr residues (the products of the PbsC-mediated Phe hydroxylation) probably does not result in two different atropisomeric structures as the rotational barrier of the biphenyl rings is likely not sufficiently high (assuming the cyclic peptide does not increase the barrier by conformational restrictions). Cyclization of two regular (not *ortho*) Tyr residues at Cε on the other hand will give rise to two atropisomeric structures, one of which is likely favored in the context of the enzyme active site, as seen for instance in the P450-catalyzed Tyr-Tyr cross-links in pyruvatides.^29^ It is possible that the less conformationally constrained cross-link of two *ortho*-Tyr residues is favorable for the bioactivity of the biphenomycins. These compounds were first isolated from at Lederle laboratories in 1967 (LL-AF283),^34^ and later also isolated from *S. griseorubiginosus* at the Fujisawa company and termed biphenomycins.^36–39^ They display potent antimicrobial activities against Gram-positive bacteria in mice by an unknown mechanism. This work shows that these compounds are RiPPs and that BGCs for analogs are widely encoded in the genomes (Figures S1 and S2). Reported total syntheses of biphenomycins required 16–22 steps.^91,92^ Deorphanizing their biosynthesis provides an alternate production strategy that may involve fermentation or chemo-enzymatic approaches, or a combination of both.

The Pbs peptides are matured by a TldD-type HExxxH-motif containing metallopeptidase PbsP to release the macrocyclic core peptide. While TldD requires a partner TldE in a heterodimeric complex for proteolytic activity,^51–53^ PbsP was active independently and a potential partner protein was not detected elsewhere in the genome of BE23. Based on the structure of biphenomycin C and the *bpm* BGC, the modified precursor BpmA is perhaps processed similarly by the PbsP homologue BpmI where the C-terminal Arg-Ser is retained. A generic carboxypeptidase may then be involved in further maturation of biphenomycin C to its A and B variants. An equivalent process could also be utilized in the C-terminal trimming of the modified Pbs peptides to release a biphenomycin B-like macrocyclic product.

The biphenomycins are examples of a growing number of RiPPs that generate one or more three-amino-acid membered rings that can be generally described as biaryl linked peptides ΩXΩ (Ω = aromatic amino acid; X = variable amino acid), or aryl-aliphatic cross-linked peptides ΩXY and YXΩ (Y = any amino acid cross-linked on an sp^3^ carbon of its side chain). These include the triceptides,^85,86^ biarylitides,^79–82,93–96^ dynobactin^97^ and darobactin.^98^ The cross-links in these and other molecules are introduced by both P450 ^80,99,100^ and rSAM enzymes^20,101^ and are diverse in structure but remarkably uniform in consisting of three amino acids. Whereas for the triceptides and biarylitides the bioactivities have not yet been elucidated and for the biphenomycins the mode of action is not known, for dynobactin and darobactin the biological activities are well understood. The cross-linked peptides generate conformationally restricted peptides that are locked in a β-strand-like mimic that interact with a β-sheet in their target.^102^ With the characterization of the biphenomycins as products of RiPP biosynthesis, this study further expands the list of such three amino acids-cross-linked natural products.

## Conclusion

This study reveals the activity of five enzymes from a BGC from . They carry out novel post-translational modifications that result in a final macrocyclic product that is structurally similar to the biphenomycins. Sequencing of the genome of a producer of biphenomycin confirmed that these long-known compounds with potent in vivo antimicrobial activity are made by a similar pathway. Thus, this work adds Phe and Tyr hydroxylation to the reactions catalyzed by MNIOs, adds B12-rSAM enzymes to the growing list of enzymes that macrocyclize peptides, and deorphanizes the biosynthesis of the biphenomycins.

## Supplementary Material



## Notes

Experimental procedures; Figures S1–S54: spectroscopic data; Tables S1–S8: gene sequences, primers, MS data, and NMR annotations (PDF)
